# Orf virus-based vectors induce potent germinal center B cell, Tfh cell, and CD8^+^ T cell responses

**DOI:** 10.1016/j.ymthe.2025.08.037

**Published:** 2025-08-27

**Authors:** Anna Lena Kastner, Melanie Müller, Anna-Friederike Marx, Mirela Dimitrova, Ingrid Wagner, Doron Merkler, Ralf Amann, Daniel D. Pinschewer

**Affiliations:** 1 Department of Biomedicine, Division of Experimental Virology, University of Basel, 4009 Basel, Switzerland; 2 Department of Immunology, Interfaculty Institute for Cell Biology, University of Tübingen, 72076 Tübingen, Germany; 3 Department of Pathology and Immunology, University of Geneva, 1211 Geneva, Switzerland; 4 Division of Clinical Pathology, Geneva University Hospital, 1206 Geneva, Switzerland

**Keywords:** vaccination, homologous prime-boost, heterologous prime-boost, Orf virus vector, parapox virus, adenovirus vector, modified vaccinia virus Ankara, B cells, follicular T helper cells, CD8^+^ T cells

## Abstract

Recombinant Orf virus (rORFV) vectors have proven safe and immunogenic in human clinical trials but the cellular correlates of antibody induction by this parapoxviral platform remain ill-defined. To characterize antigen-specific B cell responses at the cellular level, we performed adoptive transfer experiments with monoclonal B cells specific for the glycoproteins of either vesicular stomatitis virus or lymphocytic choriomeningitis virus. Immunizations of mice with rORFV delivering these glycoproteins stimulated antigen-specific B cells to engage in germinal center (GC) reactions and to differentiate into antibody-secreting cells and memory B cells. These responses could be recalled upon homologous rORFV boost. Moreover, rORFV induced antigen-specific CD8^+^ and CD4^+^ T cell responses including T follicular helper (Tfh) cells. These responses contracted over time but re-expanded upon vaccine re-administration. Pre-existing rORFV-specific anti-vector immunity interfered with CD8^+^ T cell responses to rORFV-vectored antigen, whereas CD4^+^ T cell and B cell responses were unaffected. rORFV-induced immune responses were comparable with those elicited by recombinant adenovirus- and modified vaccinia Ankara virus-based vectors, and they conferred protection against viral challenge. This study characterizes rORFV as a versatile inducer of protective GC B cell, Tfh cell, and CD8^+^ T cell responses, which can be augmented by homologous booster vaccination.

## Graphical abstract

**Figure:**
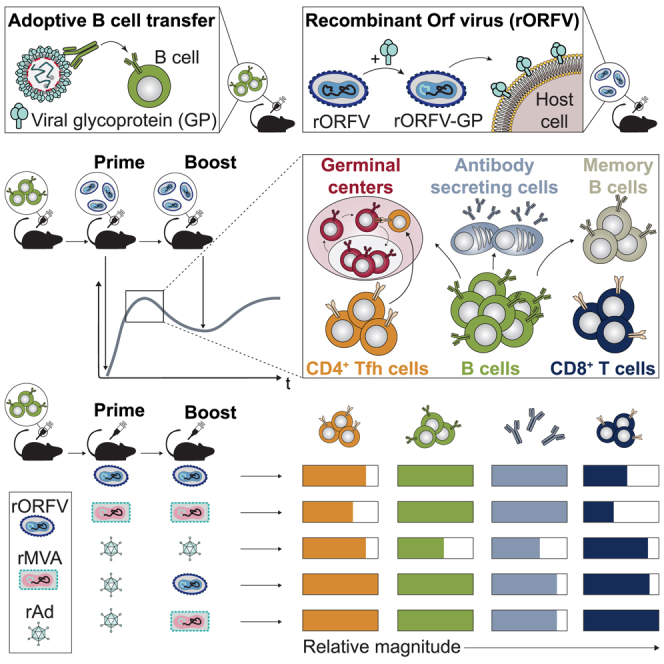

## Teaser

Performing B cell transfer studies in mice, Kastner and colleagues show that Orf virus-vectored vaccines induce potent germinal centers, follicular T helper cells, memory B cells, and plasma cells. These responses could be recalled by homologous booster vaccination and were comparable with those of adenovirus- and modified vaccinia Ankara-based vaccines.

## Introduction

The importance of vaccines for the prevention of infectious diseases and disease outbreaks has been demonstrated repeatedly, most recently during the COVID-19 pandemic.^1^ Vaccines confer protection from infection or severe disease by inducing immunological memory, which consists in pre-existing antibodies as well as in memory cells, allowing for a faster and improved response upon (re-)encounter of the corresponding pathogen. Long-lived and protective B cell memory is typically preceded by a germinal center (GC) reaction, in the context of which B cells undergo somatic hypermutation and subsequent selection, resulting in affinity maturation.^2^ B cells exit the GC to differentiate into short- and long-lived antibody-secreting cells (ASCs) as well as into memory B cells (MBCs), a complex process that remains a subject of active investigation.^3^ While long-lived ASCs continuously secrete antibodies for prolonged periods of time,^4^^,^^5^^,^^6^ providing a first line of protection upon pathogen exposure, MBCs function by mounting an accelerated secondary response.^7^ On average, the latter are of lower affinity than the former but exhibit higher cross-reactive potential than long-lived ASCs and thus are better able to protect against viral antigenic variants.^8^^,^^9^^,^^10^ An efficient B cell response, notably in terms of the GC reaction, depends on specialized cognate help from CD4^+^ T follicular helper (Tfh) cells.^11^ Thus, a vaccine aimed at eliciting humoral immune protection needs to trigger a strong B cell response alongside a robust antigen-specific CD4^+^ Tfh induction. CD8^+^ T cells, when induced in conjunction with humoral immunity, can provide an additional independent layer of antiviral protection.^12^^,^^13^^,^^14^ Viral vectors represent a promising category of vaccine delivery systems,^15^ as illustrated by the recent approval of several virally vectored vaccines, which notably comprise the vesicular stomatitis virus (VSV)-based ebolavirus (EBOV) vaccine in 2019,^16^^,^^17^ as well as several adenovirus-based (rAd) vaccines against severe acute respiratory syndrome coronavirus 2 (SARS-CoV2).^18^^,^^19^^,^^20^^,^^21^^,^^22^ While clearly a milestone in the history of virally vectored vaccines, remaining challenges call for improved vaccine platforms. Shortcomings of existing platforms for human use comprise pre-existing anti-vector immunity as prominently observed for certain rAd vectors.^23^^,^^24^^,^^25^^,^^26^ Moreover, anti-vector responses elicited upon prime vaccination can interfere with vector re-administration,^23^^,^^24^^,^^25^^,^^26^^,^^27^^,^^28^^,^^29^ requiring the combination of two different viral vectors in so-called heterologous prime-boost combinations. One example is the rAd26-based EBOV vaccine, which requires a booster vaccination with a modified vaccinia Ankara (MVA)-based vector.^30^^,^^31^^,^^32^ Downsides of heterologous prime-boost regimens consist, however, in higher complexity in the context of their development, manufacturing, regulatory approval, and eventual application in the field. Vaccine vectors with potent immunogenicity but only limited interference by pre-existing or vaccination-induced anti-vector immunity are, therefore, high in demand. Recombinant Orf virus-based vectors, which are currently tested in clinical trials for the prevention of COVID-19,^33^^,^^34^ induce only weak and/or short-lived anti-vector immune responses.^33^^,^^35^^,^^36^^,^^37^^,^^38^ The specific vector undergoing clinical development, ORFV D1701-VrV (rORFV), is derived from the Orf virus, a member of the parapoxvirus genus in the Poxviridae family.^39^ Serial passaging of Orf virus in cells of several species generated the highly attenuated rORFV, which is replication-deficient *in vivo* and apathogenic even for immunocompromised sheep, the natural host species of Orf virus.^40^^,^^41^^,^^42^ Biodistribution and shedding studies have been conducted in several species including immunocompetent and immunodeficient mice.^36^ They attested to the vector’s replication-deficient nature by documenting that vector genomes in organs approached detection limits of quantitative polymerase chain reaction within about 1 week after high-dose intravenous administration. The vector has further demonstrated a favorable safety profile in human volunteers and it can incorporate large transgenes, both important features for a vaccine vector.^36^^,^^42^^,^^43^^,^^44^ rORFV-based vectors infect predominantly antigen-presenting cells,^45^ and they were shown to induce robust cellular and humoral immune responses, conferring protection against a variety of challenge infections.^38^^,^^46^^,^^47^^,^^48^^,^^49^^,^^50^^,^^51^^,^^52^^,^^53^^,^^54^ Studies on humoral immunity were, however, limited to measurements of antibody titers in serum, and our understanding of the underlying B cell and Tfh responses are limited at best. As a parapoxvirus, ORFV exhibits only limited sequence similarity to orthopox viruses such as mpox virus, vaccinia virus, or its derivative MVA. Accordingly, there is to our best knowledge no evidence to suggest rORFV-based vectors elicit cross-protective immunity against orthopox viruses. In return, the pre-existing immunity of vaccinia virus- or MVA-vaccinated individuals is not expected to interfere with rORFV-based vaccination.

The glycoproteins (GPs) of lymphocytic choriomeningitis virus (LCMV) strain WE (WEGP) and of VSV (VSV-G) are widely used as model antigens for the study of protective antiviral B cell responses, yet exhibit vastly different immunogenicity in immunized mice.^55^^,^^56^ LCMV is a prototypic mouse model for the study of acute and chronic viral infection.^57^ The WEGP-specific pre-immune B cell repertoire of mice is limited owing to central tolerance mechanisms^58^ and a dense glycan shield, similar to the one on the envelope proteins of HIV and hepatitis C viruses, renders it a difficult target for antibody neutralization.^59^ In contrast, high-affinity VSV-G-specific B cells are abundant in the naive repertoire,^60^ and neutralizing epitopes are readily accessible to antibodies.^61^ Accordingly, VSV-neutralizing antibody (nAb) responses are mounted within 4–6 days after infection or immunization,^56^^,^^62^ whereas LCMV-nAb responses are only detectable after 60–100 days of chronic infection.^63^^,^^64^

Here, we relied on adoptive B cell transfer experiments to track and characterize antigen-specific B cells responding to rORFV vectors expressing either one of the above GPs. We found that rORFV induced not only CD8 T cells^53^ but also a strong GC B cell response alongside robust ASC as well as MBC formation, footed on a substantial antigen-specific CD4^+^ Tfh cell reaction. Anti-vector immunity did not interfere with antigen-specific B cell or CD4^+^ T cell induction, and rORFV induced protection against viral challenge.

## Results

### rORFV induces B cell and CD4^+^ T cell responses equivalent to acute LCMV infection and confers protection against chronic LCMV challenge

**Figure 1:**
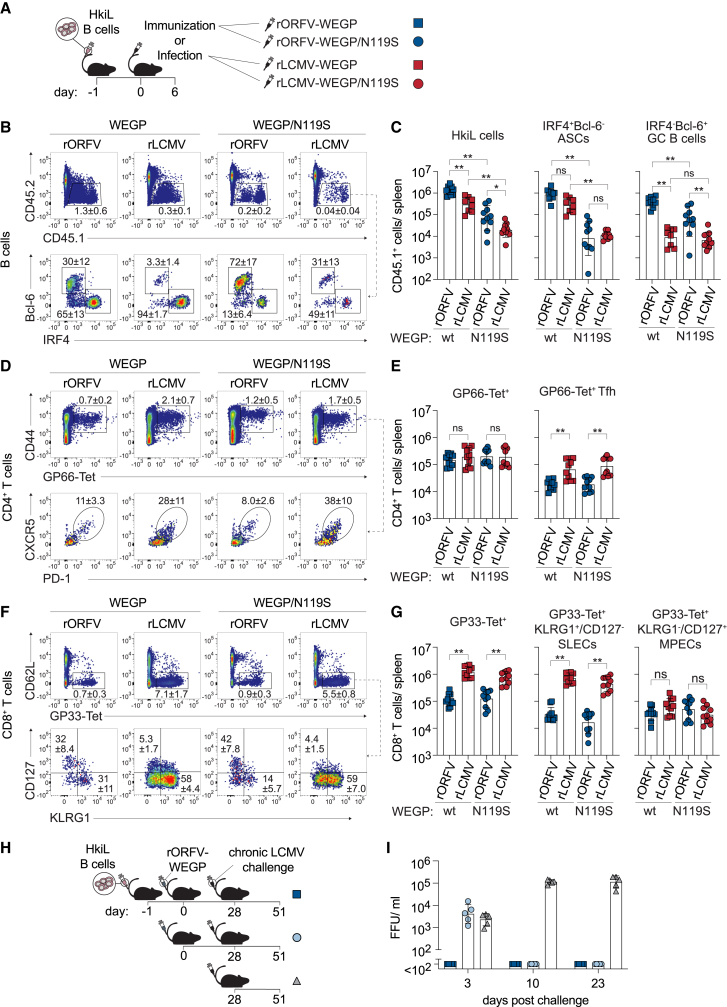
rORFV induces B cell and CD4^+^ T cell responses equivalent to acute LCMV infection and confers protection against chronic LCMV challenge (A–G) We adoptively transferred CD45.1^+^ HkiL B cells to CD45.2^+^ recipient mice on day 1 (d1) and immunized or infected them on d0 with rORFV or rLCMV encoding either WEGP or WEGP/N119S as indicated (A). Splenocytes were analyzed on d6 by flow cytometry. Representative FACS plots of adoptively transferred CD45.1^+^ HkiL B cells (B) (top row; pre-gated on live lymphocytes, see Figure S1A), with ASC (bottom row; IRF4^+^Bcl-6^–^), and GC B cell subsets (IRF4^−^Bcl-6^+^), as well as their quantification (C) on d6 p.i. Representative FACS plots of GP66-Tet^+^CD4^+^ T cells (D) (top row; pre-gated on CD4^+^CD8^−^CD19^–^ live lymphocytes, see Figure S1B) and CXCR5^+^PD1^+^ Tfh cells (bottom row; pre-gated on GP66-Tet^+^CD4^+^CD8^−^CD19^–^ live lymphocytes), as well as their quantification (E). Representative FACS plots of GP33-Tet^+^CD8 T cells (F) (top row; pre-gated on CD4^–^B220^–^ live lymphocytes, see Figure S1C) and their phenotype (bottom row; pre-gated on GP33-Tet^+^CD8^+^CD4^–^B220^–^ live lymphocytes). Absolute numbers of GP33-Tet^+^CD8 T cells (G) (left) and of the KLRG1^+^CD127^–^ SLECs and KLRG1^−^CD127^+^ MPECs contained therein (right). (H and I) We immunized two groups of mice with rORFV-WEGP on d0 (H). One of the groups had received HkiL B cells on d1 and a third group was left untreated. All groups were challenged with LCMV-Docile i.v. on d28. Viremia was monitored over time (I). Symbols in (C), (E), (G), and (I) represent individual mice (*n* = 8–10) in (C), (E), and (G) and (*n* = 5) in (I) and bars indicate the mean ± SD. The percentage of gated cells is indicated as mean ± SD. (C, E, and G) Combined data from two independent experiments analyzed by one-way ANOVA, followed by Šídák’s post-test (C) or by unpaired Student’s t test with Bonferroni correction (E and G). ∗*p* < 0.05, ∗∗*p* < 0.01; ns, not statistically significant.

To study rORFV-induced B cell responses at the cellular level, we performed adoptive transfer experiments with LCMV-specific B cells expressing as B cell receptor (BCR) the monoclonal antibody KL25 that binds WEGP at high affinity.^65^^,^^66^^,^^67^^,^^68^^,^^69^^,^^70^^,^^71^ Specifically, we used HkiL cells carrying a knockin of both the heavy and light chain V(D)J of KL25 into the respective autologous loci.^58^ Upon adoptive transfer to syngeneic CD45.2^+^ recipients these cells can be traced by means of their CD45.1 allele. We engrafted ∼500 HkiL cells per spleen and 6 days later compared the immune response induced by rORFV encoding WEGP (rORFV-WEGP) to an acute infection with LCMV Armstrong expressing the same GP (rLCMV-WEGP; Figure 1A). To assess B cell responses to both high- and low-affinity antigens, we additionally included in our study a rORFV vector (rORFV-WEGP/N119S) and an engineered variant of LCMV (rLCMV-WEGP/N119S), both carrying a point mutation at position N119S of the WEGP (WEGP/N119S). This mutation reduces the binding affinity of KL25 ∼1,000-fold,^58^^,^^72^^,^^73^ allowing us to cover in our analysis the variety in BCR affinities that is typically found in polyclonal responses.^74^ Comparing responses to the same GP, i.e., either WEGP or WEGP/N119S, respectively, HkiL cells triggered by rORFV immunization expanded to higher numbers than upon acute LCMV infection (Figures 1B, 1C, and S1A). Regardless of the virus/vector context, the high-affinity WEGP triggered a stronger expansion of HkiL cells than the low-affinity WEGP/N119S and, similarly, the number of HkiL cell-derived IRF4^+^Bcl-6^–^ ASCs was higher upon exposure of mice to high-affinity WEGP than upon encounter of low-affinity WEGP/N119S (Figures 1B and 1C). rORFV-WEGP and rORFV-WEGP/N119S induced a ∼30- and ∼10-fold more numerous GC B cell response (IRF4^−^Bcl-6^+^) than the respective rLCMV infections. LCMV infection is known to elicit high-frequency CD8^+^ and CD4^+^ T cell responses.^75^^,^^76^ Still, CD4^+^ T cells specific for the immunodominant WEGP-derived epitope GP66 (unaltered by the N119S mutation) were similarly abundant in rORFV-immunized and rLCMV-infected mice (Figures 1D, 1E, and S1B). The GP66-specific Tfh cell subset was, however, on average ∼5-fold less abundant in rORFV-immunized mice (Figures 1D, 1E, and S1B). We determined also CD8^+^ T cell responses to the immunodominant WEGP-derived epitope GP33 (unaltered by the N119S mutation), that were on average ∼8-fold higher in rLCMV-infected mice than in animals vaccinated with the rORFV vectors (Figures 1F, 1G, and S1C). When comparing GP33-specific KLRG1^+^CD127^–^ short-lived effector CD8^+^ T cells (SLECs),^77^^,^^78^ this difference amounted even to 20-fold, whereas KLRG1^−^CD127^+^ memory precursor CD8 T cells (MPECs) were similarly abundant in mice infected with rLCMV or immunized with rORFV. Taken together, these data show that rORFV-induced immunity comprises potent antigen-specific GC B cell and ASC responses accompanied by the induction of antigen-specific CD4^+^ T and Tfh cells as well as CD8^+^ T cell responses.

To assess the protective efficacy of rORFV-WEGP immunization, we challenged mice with LCMV-Docile (Figure 1H), an LCMV variant that establishes chronic infection in immunologically naive mice.^79^^,^^80^ rORFV-WEGP-vaccinated animals were either given HkiL cells 1 day prior to immunization or were left without B cell transfer. Thereby we differentiated the contribution to protection of the host-endogenous immune response from the added benefit of high-affinity HkiL cell and antibody responses. Four weeks after immunization, both vaccine groups and an additional non-vaccinated group of mice were challenged with LCMV-Docile. HkiL cell transfer with subsequent rORFV-WEGP immunization suppressed LCMV-Docile viremia to below detection limits at all time points, whereas rORFV-WEGP-vaccinated mice without HkiL B cells were viremic on day 3 after challenge but suppressed viremia to below detection limits by day 10 (Figure 1I). Non-immunized control mice remained viremic throughout the observation period of 23 days, as expected. The viral load differences on day 3 between rORFV-immunized mice with and without HkiL transfer indicated that rORFV-WEGP-induced HkiL cell responses afforded antiviral protection. Viral clearance on day 10 in rORFV-immunized mice without HkiL cell transfer was attributable to rORFV-induced endogenous immune responses with a likely contribution by CD8 T cells.^81^^,^^82^^,^^83^

### Primary and secondary immune responses to rORFV-vectored antigen of high or low affinity

**Figure 2:**
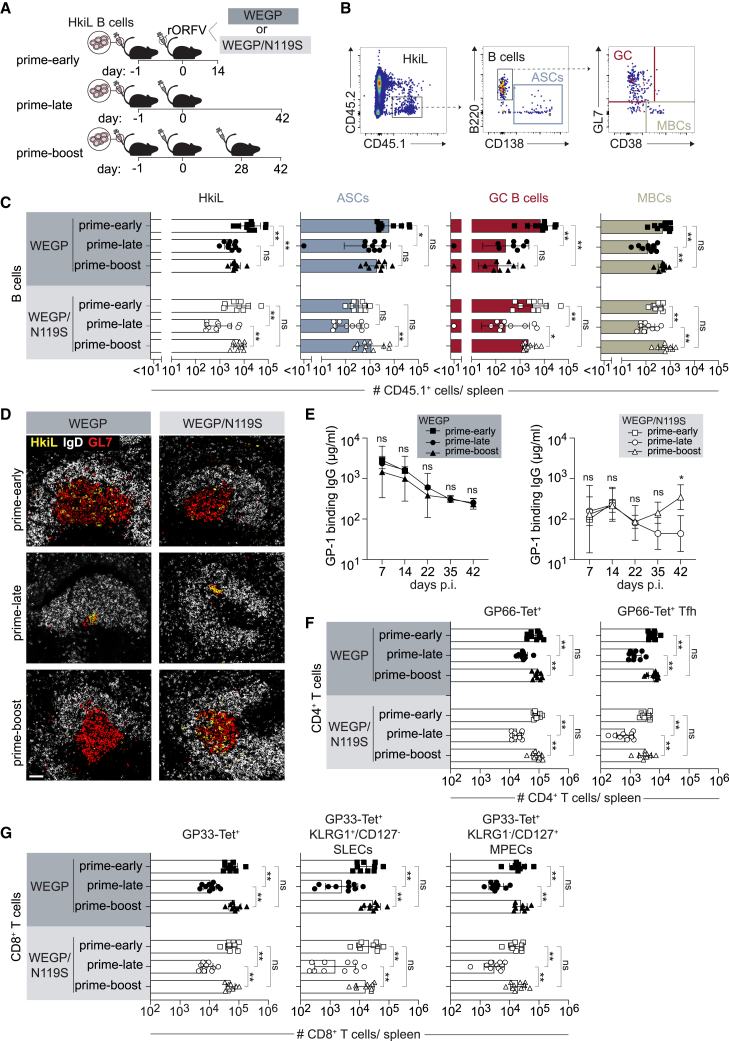
Primary and secondary immune responses to rORFV-vectored antigen of high or low affinity On d1 we transferred HkiL B cells to six distinct groups of recipient mice of which three groups were immunized with rORFV-WEGP and three with rORFV-WEGP/N119S on d0 (A). For each type of vaccination the first group of animals was analyzed on d14 (prime-early), whereas the second group of animals received a homologous booster immunization on d28 (prime-boost) and was analyzed on day 42 together with the third group that had only received a prime (prime-late). Gating strategy to analyze transferred B cells by flow cytometry (B). We quantified absolute numbers of total HkiL B cells (CD45.1^+^CD45.2^–^; pre-gated on live lymphocytes, see Figure S1A), ASCs, GC B cells, and MBCs of the different groups in spleen (C). Representative histological spleen sections (D) stained for CD45.1 (HkiL), IgD, and GL7. Scale bar, 50 μm. The concentration of WEGP domain 1 (GP-1)-specific antibody was monitored in serum over time (E). GP66-Tet^+^ CD4 T cells (F) (pre-gated on CD4^+^CD8^−^CD19^–^ live lymphocytes, see Figure S1B) and GP66-Tet^+^ Tfh cells (CXCR5^+^PD1^+^GP66-Tet^+^CD4^+^CD8^−^CD19^–^ live lymphocytes) were quantified in spleen. Numbers of GP33-Tet^+^CD8 T cells (G) (left, pre-gated on CD8^+^CD4^–^B220^–^ live lymphocytes, see Figure S1C) as well as of their KLRG1^+^CD127^–^ SLECs and KLRG1^−^CD127^+^ MPECs subsets contained therein (right). Symbols in (C), (F), and (G) represent individual mice (*n* = 9–10) and bars indicate the mean ± SD. (C, F, and G) Show combined data from two independent experiments, analyzed by one-way ANOVA followed by Tukey’s post-test. Images in (D) are representative of five mice. Symbols in (E) represent the mean of five mice and data are representative of two independent experiments. The prime-late and prime-boost groups were compared by mixed-effects analysis (WEGP groups) and by two-way ANOVA (WEGP/N119S groups) followed by Šídák’s post-test. ∗*p* < 0.05, ∗∗*p* < 0.01; ns, not statistically significant.

Prime-boost administration is a common strategy to increase the magnitude and durability of responses to vaccination.^84^ To assess primary and secondary responses to rORFV, we transferred HkiL cells to mice and administered either rORFV-WEGP or rORFV-WEGP/N119S 1 day later (Figure 2A). Subsequently, the animals were assigned to either one of three experimental groups. Mice in the prime-boost group were given a homologous booster vaccination 28 days after prime and were analyzed 14 days after the boost (42 days after prime). Mice in the prime-late group were only primed with rORFV and were analyzed simultaneously with the prime-boost group to follow the development of the primary immune response in the absence of a boost. A third group of mice (prime-early) was primed with rORFV and analyzed 2 weeks later, i.e., at an interval after prime analogous to the interval between boost and analysis of the prime-boost group. Thereby we aimed to compare the magnitude of the primary and the secondary immune response at an identical time point after vaccine administration. Within 2 weeks after rORFV-WEGP vaccination, HkiL cells had differentiated into substantial ASC, GC, and MBC populations, as expected (Figures 2B, 2C, and S2A; compare Figures 1B and 1C). As evident from the comparison of the prime-late to the prime-early group, these responses contracted over time, which was most pronounced for GC B cells and MBCs. Interestingly, booster immunization augmented the HkiL MBC population but failed to increase the corresponding GC or ASC counts (comparing prime-boost withg prime-late). In accordance with these flow cytometry results, immunofluorescence analyses of spleen sections identified HkiL cells in GCs of prime-early mice (Figures 2D and S3). By the prime-late time point, these GCs had contracted considerably in size, but HkiL cells persisted therein. Interestingly, GCs were much larger in the prime-boost than the prime-late group suggesting re-expansion after boost, but the latter GCs were virtually devoid of HkiL cells, indicating that HkiL cells were not effectively recruited into and/or retained in the GC response to rORFV-WEGP boost. Analogously to the above high-affinity vaccination setting, the primary response to rORFV-WEGP/N119S contracted over time (comparing prime-late with prime-early; Figures 2C and S2A). Unlike upon high-affinity rORFV-WEGP re-administration, however, booster immunization with the low-affinity rORFV-WEGP/N119S induced a significant secondary expansion of the total HkiL B cell progeny. This secondary expansion was not only driven by an increase in ASCs and MBCs (comparing prime-late with prime-boost), but also the HkiL GC response was restored to prime-early levels (comparing prime-boost with prime-early; Figure 2C and S2A). Accordingly, immunofluorescence analysis of the spleen evidenced a fair representation of HkiL cells in re-expanded GCs of rORFV-WEGP/N119S-primed and -boosted mice (Figures 2D and S3). Antibody responses of HkiL cells responding to high-affinity rORFV-WEGP prime vaccination were higher than those to rORFV-WEGP/N119S prime (Figure 2E). In keeping with the finding that rORFV-WEGP/N119S boost but not rORFV-WEGP re-vaccination augmented HkiL ASC numbers, the former but not the latter resulted in a significant augmentation of WEGP-specific antibody responses (compare prime-boost with prime-late). HkiL cells can hypermutate to improve recognition of their low-affinity ligand WEGP/N119S.^58^ Using an enzyme-linked immunosorbent assay (ELISA) surrogate of serum antibody affinity to WEGP/N119S, we found that rORFV-WEGP/N119S prime-only as well as prime-boost immunization resulted in affinity maturation of HkiL cells over time (Figure S2D). Additionally, we enumerated GP66-specific CD4^+^ T cells and GP33-specific CD8^+^ T cells using MHC class I and MHC class II tetramers, respectively (Figures 2F, 2G, S2B, and S2C). As expected (compare Figures 1D–1G), these responses were unaffected by the N119S mutation. GP66-specific total CD4^+^ T cells and also the Tfh subset contracted over time but re-expanded after the boost (Figure 2F), and an analogous pattern was observed for GP33-specific CD8^+^ T cell responses (Figure 2G). SLECs as well as MPEC counts followed this pattern of contraction and re-expansion. Hence, rORFV booster vaccination lifted T cell and MBC responses back to the levels reached on day 14 after prime, yet failed to expand them significantly above those levels. HkiL ASC and GC B cell responses were only augmented in the context of low-affinity rORFV-WEGP/N119S vaccination but not upon high-affinity rORFV-WEGP re-administration.

### Anti-vector immunity impairs CD8^+^ T cell responses to rORFV-vectored antigen, whereas CD4^+^ T cell and B cell responses are unaffected

**Figure 3:**
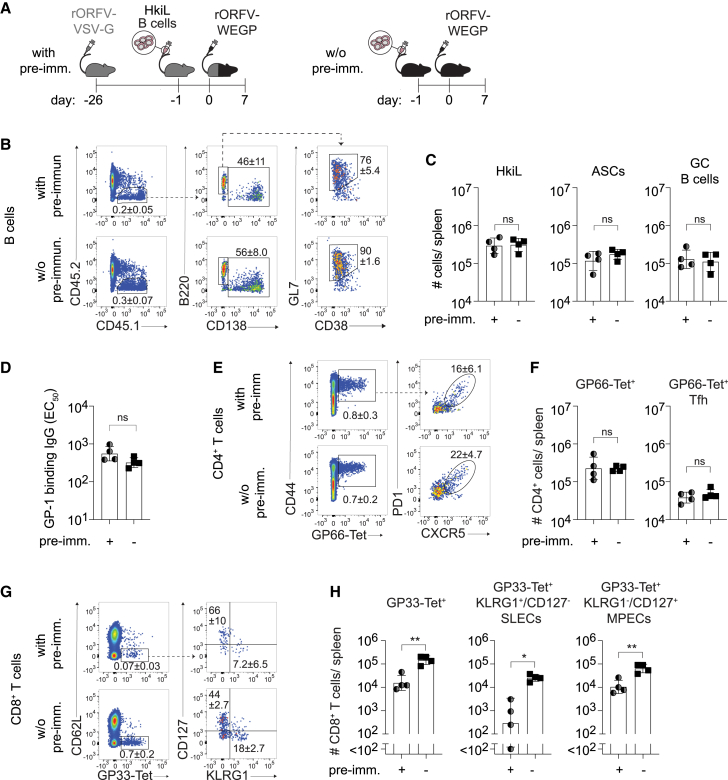
Anti-vector immunity impairs CD8^+^ T cell responses to rORFV-vectored antigen whereas CD4^+^ T cell and B cell responses are unaffected We pre-immunized one group of mice with rORFV-VSV-G (with pre-imm.) on d26 and on d1 we transferred HkiL B cells to the pre-immunized mice as well as to a second untreated control group (w/o pre-imm.) (A). Both groups were immunized with rORFV-WEGP on d0 and splenocytes were analyzed on d7 by flow cytometry. Representative FACS plots of adoptively transferred CD45.1^+^ HkiL B cells (B) (pre-gated on live lymphocytes, see Figure S1A) with ASC (CD138^+^) and GC B cell subsets (B220^+^CD138^−^GL7^+^CD38^–^) as well as their quantification (C). GP-1 binding serum antibody titers on d7 (D). Representative FACS plots of GP66-Tet^+^CD4 T cells (E) (left, pre-gated on CD4^+^CD8^−^CD19^–^ live lymphocytes, see Figure S1B) and CXCR5^+^PD1^+^ Tfh cells (right, pre-gated on GP66-Tet^+^CD4^+^CD8^−^CD19^–^ live lymphocytes), as well as their quantification (F). Representative FACS plots of GP33-Tet^+^CD8 T cells (G) (left, pre-gated on CD4^–^B220^–^ live lymphocytes, see Figure S1C) and their phenotype (right, pre-gated on GP33-Tet^+^CD8^+^CD4^–^B220^–^ live lymphocytes). Absolute numbers of GP33-Tet^+^CD8 T cells (H) (left) and of the KLRG1^+^CD127^–^ SLECs and KLRG1^−^CD127^+^ MPECs subsets contained therein (right). Symbols in (C), (D), (F), and (H) represent individual mice (*n* = 4) and bars indicate the mean ± SD. The percentage of gated cells is indicated as mean ± SD. Data in (B)–(H) are representative of two independent experiments and were analyzed by unpaired Student’s t tests (C, D, F, and H). ∗*p* < 0.05, ∗∗*p* < 0.01; ns, not statistically significant.

The observation that secondary B cell and T cell responses to rORFV immunization failed to exceed the primary response (compare Figure 2) prompted us to test whether anti-vector immunity impeded homologous rORFV prime-boost. To induce anti-vector immunity without concomitant elicitation of LCMV-GP-specific immune responses, we immunized mice with rORFV encoding the GP of the antigenically unrelated VSV (rORFV-VSV-G; Figure 3A). Twenty-five days later we adoptively transferred HkiL B cells to these rORFV-pre-immune mice and simultaneously also to an immunologically naive control group of animals, followed by rORFV-WEGP vaccination the consecutive day. We failed to detect significant differences in the overall magnitude of HkiL B cell responses (Figures 3B and 3C), and also these cells’ differentiation into ASCs and GC B cells was unaffected by rORFV-VSV-G pre-immunization. Moreover, HkiL B cell-derived GP-1-specific antibody titers were comparable in the two groups (Figure 3D). rORFV-VSV-G pre-immunization did not affect the magnitude of GP66-specific total CD4^+^ T cell or Tfh cell counts either (Figures 3E and 3F). In marked contrast, however, GP33-specific CD8^+^ T cells were reduced in pre-immunized mice, with SLEC numbers most affected, but also MPECs were reduced (Figures 3G and 3H). In summary, our data demonstrate that CD8^+^ T cell induction but neither B cell nor CD4^+^ T cell responses to rORFV-vectored immunization were negatively impacted by pre-existing anti-vector immunity.

### Primary and secondary B cell and CD4^+^ T cell responses to rORFV-vectored VSV-G

**Figure 4:**
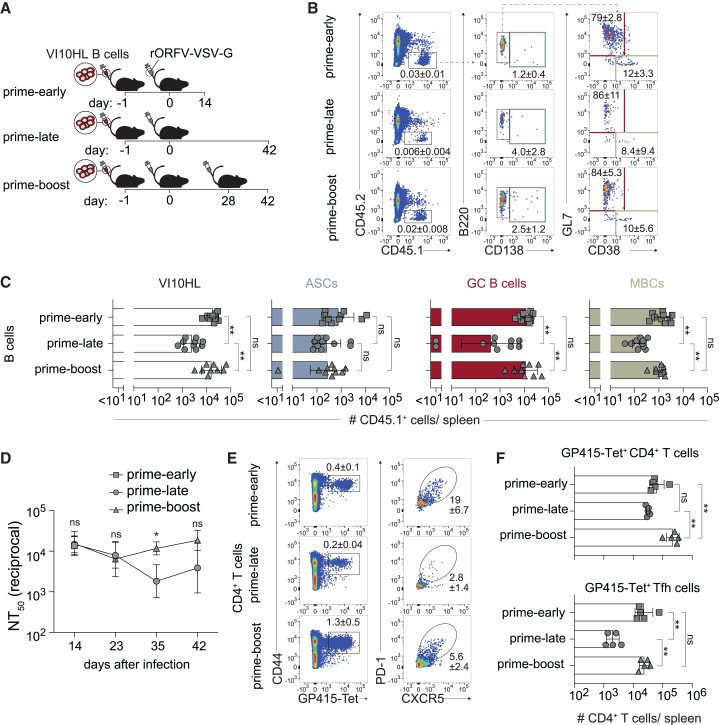
Primary and secondary B cell and CD4^+^ T cell responses to rORFV-vectored VSV-G We transferred VI10HL B cells to three groups of recipient mice on d1 and immunized them with rORFV-VSV-G on d0 (A). Splenocytes of the first group were analyzed 14 days after prime immunization (prime-early) by flow cytometry. A second group received a homologous booster immunization on d28 (prime-boost) and was analyzed on d42 together with the third group, which only received a prime immunization (prime-late). Representative FACS plots of CD45.1^+^VI10HL cells (B) (pre-gated on live lymphocytes, see Figure S1A), ASCs, GC B cells, and MBCs in spleen and their quantification (C). VSV-neutralizing antibody titers were monitored in serum over time (D). Representative FACS plots of GP415-Tet^+^CD4 T cells (E) (left, pre-gated on CD4^+^CD8^−^CD19^–^ live lymphocytes, see Figure S1B) and GP415-Tet^+^ Tfh cells (right, pre-gated on G415-Tet^+^CD4^+^CD8^−^CD19^–^ live lymphocytes) and their quantification (F) in spleen. Symbols in (C) and (F) represent individual mice (*n* = 9–10) in (C) and (*n* = 5) in (F) and bars indicate the mean ± SD. Symbols in (D) represent the mean of five mice. The percentage of gated cells is indicated as mean ± SD. Data in (C) are combined from two independent experiments and in (D and F) are representative of two independent experiments. (C and F) were analyzed by one-way ANOVA followed Tukey’s post-test. Groups prime-late and prime-boost in (D) were compared by two-way ANOVA followed by Šídák’s post-test. ∗*p* < 0.05, ∗∗*p* < 0.01; ns, not statistically significant.

Besides the vector backbone also the vectorized antigen can shape vaccine responses.^56^ To validate and generalize our findings with LCMV-GP-expressing rORFV vectors, we extended our studies to B cell responses against rORFV-vectored VSV-G, an antigen with immunogenic properties that are vastly different from LCMV-GP.^55^ Congenitally labeled (CD45.1^+^) VI10HL B cells,^58^ expressing a monoclonal VSV-G-specific BCR, were adoptively transferred to CD45.2^+^-recipient mice, which were subsequently immunized with rORFV-VSV-G (Figure 4A). To assess primary and secondary responses to rORFV-VSV-G, animals were assigned to experimental groups analogous to the experiments in Figure 2 (prime-early, prime-late, and prime-boost). Upon rORFV-VSV-G immunization, VI10HL B cells differentiated into GCs, MBCs, and ASCs, as observed for immunizations with rORFV-WEGP (Figures 4B and 4C; compare Figures 2B and 2C). These responses contracted from day 14 onward, which was most pronounced for GC B cells and MBCs (compare prime-early with prime-late). Homologous booster vaccination afforded an overall increase in total VI10HL B cell progeny (CD45.1^+^), driven by GC B cell and MBC counts that were back up in the range they had been after prime (compare prime-early with prime-boost). In contrast, VI10HL ASC numbers did not measurably benefit from booster vaccination (Figure 4C). VSV-G-specific nAb titers were back in the range they had been on day 14 after prime (Figure 4D), recapitulating the response pattern of rORFV-WEGP/N119S prime-boost. Unlike in the latter experimental setting, where GP-1-specific antibody responses are virtually exclusively HkiL cell derived, both VSV-G-specific endogenous B cells and adoptively transferred VI10HL cells may have contributed to increased VSV-G-specific antibody titers after boost. Using MHC class II tetramer we monitored also rORFV-induced CD4^+^ T cell responses specific to the immunodominant VSV-G epitope GP415 (Figures 4E and 4F). This response did not significantly contract after initial expansion (compare prime-early with prime-late) and subsequent to boost expanded to frequencies that exceeded those in the prime-early group. GP415-specific Tfh cells were also detected and in contrast to GP415-specific CD4^+^ T cell responses contracted ∼10-fold from day 14 to day 42 (compare prime-early with prime-late). Homologous booster vaccination restored prime-early levels of GP415-specific Tfh responses. Taken together, the assessment of rORFV-VSV-G in the context of VI10HL cell transfer corroborated our finding that rORFV vectors induce robust B cell and CD4^+^ T cell responses comprising a substantial Tfh cell component.

### rORFV immunization induces durable protective antibody immunity against lethal VSV challenge

**Figure 5:**
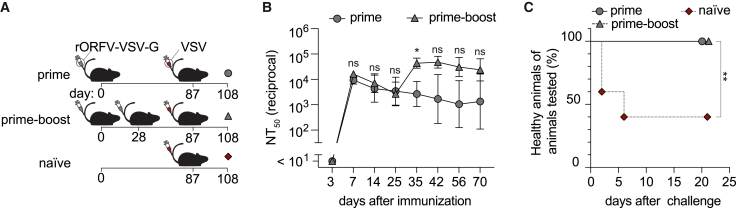
rORFV immunization induces durable protective antibody immunity against lethal VSV challenge We immunized mice with rORFV-VSV-G on d0 (A) (prime), on d0 and d28 (prime-boost), or left them untreated (naive). All groups were challenged with VSV i.v. on d87. VSV-neutralizing antibody titers were followed over time (B). We monitored immunized (combine prime and prime-boost groups) as well as naive mice for signs of disease after VSV challenge and euthanized mice reaching the humane endpoint (C). Symbols in (B) represent the mean of five mice per group and 100% in (C) equals 5 naive mice and 10 immunized mice, respectively. Data in (B) were analyzed by mixed-effects analysis followed by Šídák’s post-test. Kaplan-Meier survival curves of immunized and naive mice in (C) were analyzed by the Mantel-Cox test. ∗*p* < 0.05, ∗∗*p* < 0.01; ns, not statistically significant.

Durable antibody titers represent a key correlate of protection for many clinically used vaccines. Thus, we assessed the longevity of the polyclonal antibody response upon immunization with rORFV-VSV-G (Figures 5A and 5B). After single-dose rORFV-VSV-G immunization, VSV-nAb titers declined less than 10-fold over an observation of >70 days. In keeping with the observations made in animals given VI10HL cell transfer (compare Figure 4D), rORFV-VSV-G-induced antibody titers were augmented when mice were given a homologous booster vaccination on day 28 after prime (Figure 5B). Protection of immune mice against lethal VSV infection is antibody mediated.^85^ When challenged with a lethal dose of VSV on day 87 after rORFV-VSV-G prime or on day 59 after homologous boost, respectively, the animals were uniformly protected against VSV-induced paralytic central nervous system disease (Figure 5C).^55^ In contrast, three out of five unvaccinated control mice (naive) reached humane endpoints and had to be euthanized within 1 week after challenge. These results confirmed the ability of rORFV to induce long-lasting and protective antibody immunity.

### Comparison of rORFV to vaccine vectors in clinical use and its combination therewith in heterologous prime-boost vaccination

**Figure 6:**
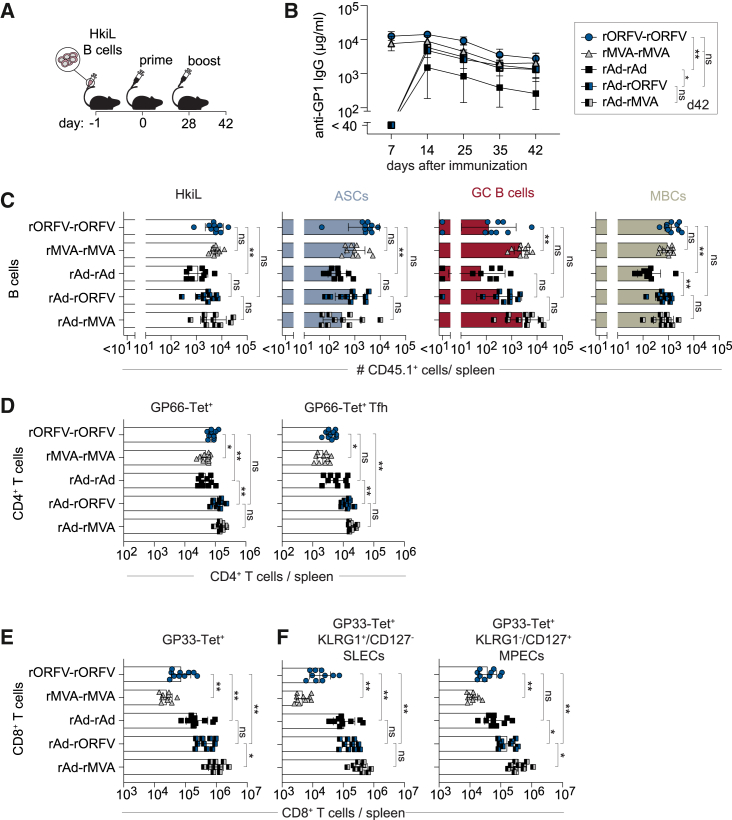
Comparison of rORFV to vaccine vectors in clinical use and its combination therewith in heterologous prime-boost vaccination We adoptively transferred HkiL B cells to recipient mice on d1 and immunized them on d0 with rORFV, rMVA, or rAd encoding WEGP (A). On d28, three groups of mice were boosted in a homologous manner with the respective vector. An additional two groups of rAd-WEGP-primed mice were boosted with rORFV-WEGP or rMVA-WEGP, respectively, as indicated in the chart in (B). GP1-binding serum antibody titer over time (B). We determined CD45.1^+^ HkiL cell numbers (C) (pre-gated on live lymphocytes; see Figure S1A) and the subsets of ASCs (CD138^+^), GC B cells (GL7^+^CD38^–^), and MBCs contained therein (GL7^−^CD38^+^) by flow cytometry on d42 in spleen. Absolute numbers of GP66-Tet^+^CD4 T cells (D) (left, pre-gated on CD4^+^CD8^−^CD19^–^ live lymphocytes; see Figure S1B) and CXCR5^+^PD1^+^ Tfh cells (right, pre-gated on GP66-Tet^+^CD4^+^CD8^−^CD19^–^ live lymphocytes). GP33-Tet^+^CD8 T cell numbers (E) (pre-gated on CD4^–^B220^–^ live lymphocytes, see Figure S1C) and the KLRG1^+^CD127^–^ SLEC and KLRG1^−^CD127^+^ MPEC subsets contained therein (F). Symbols in (B) represent the mean of five mice. Symbols in (C)–(F) represent individual mice (*n* = 10) and bars indicate the mean ± SD. Data in (B) are representative of two independent experiments and the d42 time point was analyzed by one-way ANOVA followed by Šídák’s post-test. (C–F) Show combined data from two independent experiments analyzed by one-way ANOVA followed by Šídák’s post-test. ∗*p* < 0.05, ∗∗*p* < 0.01; ns, not statistically significant.

We aimed to compare rORFV-induced immune responses to those elicited by rMVA- or rAd-vectored vaccines as prototypic examples of clinically used viral vaccine vector systems. To this end we transferred HkiL B cells to recipient mice 1 day prior to immunization with rMVA, rAd, or rORFV encoding WEGP (Figure 6A). For each vector, one group of mice was administered a homologous booster vaccination 28 days later, followed by analysis on day 42 of the experiment. Additionally, we included a group of animals given rAd prime and rMVA boost (rAd-rMVA), representing a well-established combination of vectors that is in clinical use for the prevention of Ebola hemorrhagic fever.^30^^,^^31^^,^^32^ Finally, we were interested to test whether rORFV would effectively substitute for rMVA, also a poxviral vector, as a booster of rAd-primed immune responses (rAd-rORFV). rORFV and rMVA immunization elicited rapid WEGP-specific antibody responses, which were close to their maximum already on day 7, whereas the response to rAd prime was only detected on day 14 after vaccination (Figure 6B). Antibody titers on day 42 after homologous rORFV vaccination (rORFV-rORFV) were at least equivalent to those elicited by the other immunization regimens tested, and they were significantly higher than those induced by rAd-rAd. HkiL B cell progeny numbers on day 42 after rORFV-rORFV vaccination were comparable with those elicited by rMVA-rMVA, again outperforming rAd-rAd (Figures 6C and S4A). The same hierarchy pattern was also evident at the level of HkiL ASC and MBC numbers, whereas rMVA elicited higher HkiL GC B cell numbers than rORFV-rORFV. HkiL B cell responses to rAd-rORFV and rAd-rMVA were of comparable magnitude in terms of ASC, GC B cell, and MBC progeny numbers, suggesting that these two heterologous prime-boost regimens were equivalent for the induction of B cell responses. All vector combinations elicited robust GP66-specific CD4^+^ T cell responses (Figure 6D; Figure S4B). Still, homologous rORFV-rORFV immunization induced significantly more GP66-specific CD4^+^ T cells than rMVA-rMVA or rAd-rAd. GP66-specific CD4^+^ T cell responses to rORFV-rORFV and rAd-rORFV immunization were in comparable ranges, while the latter exceeded the one induced by homologous rAd-rAd. In terms of GP66-specific Tfh responses, heterologous rAd-rORFV immunization outperformed homologous rAd-rAd and rORFV-rORFV. Heterologous rAd-rORFV and rAd-rMVA induced comparable GP66-specific total CD4^+^ T cell and Tfh cell responses. In keeping with the well-documented ability to induce high-frequency CD8^+^ T cell responses,^86^^,^^87^^,^^88^^,^^89^^,^^90^ rAd homologous prime-boost induced GP33 responses exceeding those to rORFV-rORFV by ∼4-fold (Figures 6E and S4C). rAd-rORFV heterologous prime-boost elicited ∼6-fold higher splenic GP33-specific CD8^+^ T cell counts than rORFV-rORFV, but these responses were not significantly different from rAd-rAd. rAd-rMVA induced the highest responses, exceeding those to rAd-rAd by ∼3-fold. When assessing GP33-specific SLEC and MPEC counts, we found similar hierarchies and differences as observed for total GP33-specific CD8^+^ T cells (Figures 6F and S4C).

Taken together, these results indicate that rORFV-induced B cell as well as CD4^+^ and CD8^+^ T cell responses are in similar ranges as those elicited by rMVA and rAd, and that rORFV can effectively be combined with rAd-based vaccine vector technology for use in heterologous prime-boost combinations.

## Discussion

This study provides a detailed analysis of the B cell response to immunization with rORFV, alongside with the analysis of CD4^+^ and CD8^+^ T cell immunity. While corroborating earlier reports that rORFV induces strong antibody responses,^33^^,^^38^^,^^46^^,^^47^^,^^48^^,^^49^^,^^50^^,^^51^^,^^52^^,^^54^ we show that rORFV elicits potent B cell responses that comprise substantial GC reactions, MBCs as well as ASCs. The latter seem long-lived as evident from the durability of antibody titers and protective antibody immunity against a lethal VSV challenge. Equally importantly, rORFV elicits antigen-specific CD8^+^ T cells and induces CD4^+^ T cell responses comprising a substantial Tfh subset as a source of B cell help.

As judged from the comparison with LCMV infection and to vaccination with rAd or rMVA, rORFV is a strong inducer of B cell and CD4^+^ T cell responses. Furthermore, rORFV demonstrated significant potential for use in homologous prime-boost vaccination. This feature is expected to facilitate product development, manufacturing, and roll-out of the vector in the field. The mechanisms whereby anti-vector immunity interferes with CD8^+^ T cell induction by rORFV awaits experimental investigation. In orthopox- and arenavirus-vectored immunization, vector backbone-specific CD8^+^ T cell responses suppress CD8^+^ T cell responses to vectorized cargo owing to clonal competition and immunodominance.^91^^,^^92^ For rAd-based vectors, it has been shown that vector-specific antibodies limit the duration of vectored transgene expression, which is mediated by antibody effector mechanisms rather than by prevention of vector entry into target cells.^93^^,^^94^ Analogously, the failure to detect vector-neutralizing antibodies in rORFV-vaccinated individuals^33^ suggests that prevention of vector entry is not a main mechanism whereby pre-existing anti-ORFV immunity interferes with CD8^+^ T cell induction. In the absence of experimental evidence it seems likely that mechanisms such as clonal competition and/or the accelerated elimination of rORFV account for the interference of anti-vector immunity with CD8^+^ T cell induction by the same. The failure to detect analogous effects of pre-existing anti-vector immunity on CD4^+^ T cell and B cell responses may be due to less stringent requirements in terms of antigen amounts and duration of antigen production. In line with this hypothesis CD4^+^ T cells are known to be more sensitive to stimulation by residual low-level antigen than CD8^+^ T cells,^95^ and antigen stored on follicular dendritic cells can sustain B cell responses for prolonged periods of time.^96^ Related to the above, high-frequency CD8^+^ T cell responses were best induced in heterologous prime-boost combinations. The equivalence of heterologous rAd-rORFV immunization to the widely used rAd-rMVA regimen, at times perceived as a golden standard in the field, is noteworthy and shows the utility of rORFV beyond stand-alone applications. Heterologous prime-boost regimens combining rORFV with vectors other than rAd5 should be evaluated in future work to potentially select vectors less affected by pre-existing immunity.^23^^,^^24^^,^^25^^,^^26^^,^^28^^,^^29^

When studying HkiL and VI10HL B cells in the context of rORFV immunization we noticed that their secondary expansion failed to exceed the primary response. Our interference studies suggested, however, that limited secondary responses were unlikely due to anti-vector immunity. Hence, we suspect this response pattern may relate to intrinsic features of our adoptive B cell transfer setting, in which specific naive B cells are supplied prior to prime but not again prior to boost. Evidence has emerged in recent years that only a minor proportion of MBCs re-enter GCs upon recall.^97^ Accordingly, a majority of the secondary GC consists in newly recruited, formerly naive B cells, offering a plausible explanation for the relatively inefficient recall of HkiL and VI10HL B cells in our prime-boost setting. We further observed that re-administration of rORFV-WEGP/N119S induced a re-expansion of antigen-specific B cells to levels equivalent to the primary response, which was not the case for rORFV-WEGP. The HkiL BCR exhibits ∼1,000-fold higher affinity for WEGP than for WEGP/N119S,^58^ which can likely explain the lack of re-expanding B cells after re-administration of rORFV-WEGP. High-affinity B cells exhibit a higher propensity to differentiate into ASCs than low-affinity B cells.^98^^,^^99^ Accordingly, rORFV-WEGP immunization induced more HkiL ASCs and higher specific antibody titers than rORFV-WEGP/N119S, which in return is expected to result in more pronounced antibody feedback.^100^^,^^101^^,^^102^ While this regulatory mechanism supposedly serves to ascertain a balanced response to several epitopes of a given antigen,^101^ our HkiL B cell transfer system is characterized by a general paucity of endogenous B cell clones reactive to the WEGP subunit (GP1) due to central tolerance mechanisms.^58^ In this context, antibody feedback may suppress rather than broaden the antibody response, an effect that, in keeping with the observed pattern, should be more pronounced for rORFV-WEGP owing to higher antibody titers after prime immunization. Analogously to rORFV-WEGP/N119S, we observed that re-administration of rORFV-VSV-G induced a re-expansion of adoptively transferred B cells. In contrast to the HkiL B cell transfer system, however, VSV-G-specific B cells are abundant in the naive polyclonal repertoire^60^ and the serum antibody response consists of both VI10HL cell as well as host-endogenous antibody responses, complicating the interpretation of potential antibody feedback effects on the VI10HL B cell response.

In summary, our study provides a detailed characterization of the cellular basis for humoral immune responses to rORFV vaccination. It positions rORFV as a vector with several advantageous features and an immunogenicity profile that is on a par with or superior to more commonly used viral vector technologies.

### Limitations of the study

This study has limitations in that epitope-specific Tfh cell responses were identified by MHC class II tetramers in conjunction with surface markers allowing for their enumeration over time, but more detailed functional and/or transcriptional characterization of these responses such as the level of expression of the signature cytokine IL-21 should be the subject of future studies. Moreover, the adoptive transfer of monoclonal B cells expressing a very high-affinity BCR for WEGP and VSV-G, respectively^60^^,^^73^ has precluded an assessment of antibody affinity maturation in experiments where these antigens were vectorized.

## Materials and methods

### Animals and ethics statement

C57BL/6J WT mice were initially purchased from Charles River and were further bred at the Laboratory Animal Science Center (LASC) of the University of Zurich and at the ETH Phenomics Center (EPIC). HkiL,^58^ VI10HL,^58^ and TgL^64^^,^^71^ mice have been described and were bred at EPIC. All mice were bred and experiments were conducted under specific pathogen-free conditions. Within experiments mice were age and sex matched. However, both genders were used to reduce animal numbers bred for research purposes. All experiments were performed at the University of Basel in accordance with the Swiss law for animal protection and with authorization from the Cantonal veterinary office. Experimental groups were not randomized and the experiments were not conducted in a blinded fashion.

### Cell lines

We obtained NIH3T3cells from ATCC (CRL-1658) and cultured them in DMEM (Sigma, D0819; supplemented with 10% fetal calf serum [FCS]). BHK21 cells were purchased from ECACC (85011433) and cultured in DMEM (supplemented with 10% FCS, 10 mM HEPES [Gibco, 15630056], 1 mM Na-pyruvate [Gibco, 11360070], and 0.3 g/L tryptose phosphate broth [Sigma, T8782]). Vero cells were obtained from ATCC (CCL-81) and VeroE6 cells were purchased from ECACC (85020206) and cultured in in DMEM (supplemented with 10% FCS). All cells were cultured at 37°C in an atmosphere of 5% CO_2_ and were confirmed negative for mycoplasma at regular intervals.

### Viruses, virus titrations, immunizations, and challenge infections

The rORFV vectors expressing either the wild-type GP of the LCMV strain WE (WEGP) (rORFV-WEGP), the N119S mutant of the WEGP (rORFV-WEGP/N119S), or the GP of VSV (rORFV-VSV-G) were generated as follows. The genes of WEGP, WEGP/N119S, and VSV-G were synthesized by Gene Art (ThermoFisher Scientific) and cloned into plasmid pV-Cherry.^43^ The correct insertion and sequences were confirmed by restriction digest and sequencing (Eurofins Genomics). The resulting transfer plasmids pV-LCMV-WEGP, pV-LCMV-WEGP/N119S, and pV-VSV-G were used to transfect Vero cells (infected with D1701-VrV-GFP-D12-Cherry using the SF Cell Line 4D-Nucleofector X Kit [Lonza]) to replace the encoded green fluorescent protein (GFP) gene by homologous recombination as described previously.^43^^,^^103^ The generated ORFV recombinants D1701-VrV-LCMV-WEGP-D12-Cherry (rORFV-WEGP), D1701-VrV-LCMV-WEGP/N119S-D12-Cherry (rORFV-WEGP/N119S), and D1701-VrV-VSV-G-D12-Cherry (rORFV-VSV-G) were selected via fluorescence-activated cell sorting (FACS) using a SH800S Cell Sorter (Sony Biotechnology, Bothell, WA). Their identification was confirmed by polymerase chain reaction using insert- and locus-specific primers. Propagation and purification of ORFV recombinants followed established protocols.^103^ To assess the genetic stability of the inserted proteins, 10 serial passages were conducted in Vero cells. Virus titers were determined using a standard plaque assay.^43^

The recombinant E1-deleted adenovirus (rAd) type 5 vector expressing the WEGP (rAd-WEGP)^104^ was grown and purified as described^105^ and has been generously provided by Florian Kreppel, University Witten/Herdecke, Germany. The recombinant MVA encoding the WEGP (rMVA-WEGP) was kindly provided by Thomas Brocker, LMU, Germany, and had originally been produced by Gerd Sutter. The LCMV substrain Docile^80^ was originally obtained from Rolf Zinkernagel and Hans Hengartner, University of Zurich, Switzerland. Recombinant LCMV strain Armstrong expressing the WEGP (rLCMV-WEGP)^106^ or the N119S mutant (rLCMV-WEGP/N119S) as well as VSV serotype Indiana^107^ were grown on BHK21 cells at multiplicity of infection of 0.01. The VSV∗ΔG (Luc) was generously provided by Gert Zimmer, University of Bern, Switzerland, and has been generated as described previously.^108^ rORFV vectors and rMVA-WEGP were administered intravenously (i.v.) into the tail vein at a dose of 1 × 10^7^ PFU per mouse. rAd-WEGP was administered i.v. at a dose of 5 × 10^8^ VP per mouse. rLCMV was administered i.v. at a dose of 200 FFU to elicit an acute infection. To challenge mice with LCMV-Docile, we administered 2 × 10^6^ FFU i.v. per mouse, inducing a chronic infection. For lethal challenge with VSV we administered a dose of 3 × 10e^8^ PFU per mouse i.v., which in non-immune mice can induce infection of the spinal cord. Mice were monitored daily for signs of disease manifesting as ascending hindlimb paralysis and animals were euthanized at the onset of disease (humane endpoint).

To determine LCMV viremia, we collected one drop of blood into 950 μL of balanced salt solution (BSS) supplemented with 1 IE/mL heparin-Na (B. Braun, B01AB01). Viremia samples and LCMV virus stocks were titrated with a focus forming assay adapted from Battegay et al.^109^ After preparing serial dilutions of virus stocks or of blood samples in 96-well plates in MEM (Sigma, M0446; supplemented with 2% FCS and 1% penicillin/streptomycin [P/S]; Thermo Fisher Scientific, 15140-122), we added 3 × 10^4^ 3T3 cells to each well and incubated the cells with virus for 2–3 h at 37°C and 5% CO_2_. Next, we added overlay (1% methylcellulose, 10% FCS, 1% P/S in DMEM) and incubated the assay for 48 h at 37°C and 5% CO_2_. We fixed the cells by adding 4% paraformaldehyde (PFA) (Sigma, 8187151000) in phosphate-buffered saline (PBS) for 10 min at room temperature (RT). Next, we permeabilized the cells with 1% Triton X-100 (Sigma, T8787) in balanced salt solution (BSS) for 20 min at RT. By adding 5% FCS in PBS for 30 min we blocked unspecific binding and then added the rat anti-LCMV-NP antibody VL4^109^ in PBS containing 2.5% FCS for 1 h at RT. We washed the plates with tap water and incubated them with the secondary horseradish peroxidase (HRP)-conjugated goat anti-rat IgG antibody (Jackson ImmunoResearch, 112-035-003) diluted 1:500 in PBS containing 2.5% FCS for 1 h at RT. Plates were washed and infectious foci were visualized by a color reaction using 1.4 mM 3,3′-diaminobenzidine tetrahydrochloride hydrate (Sigma, D-5637), 0.5 g/L ammonium nickel(II)sulfate hexahydrate (Sigma, 09885), and 0.015% H_2_O_2_ (Sigma, 216763) in PBS and quantified by using an Immunospot S6 device (C.T.L.).

### Adoptive B cell transfer

For adoptive B cell transfer, we mechanically disrupted spleens from HkiL or VI10HL mice to obtain single cell suspensions. Next, we isolated B cells from the suspension by using the EasySep mouse B cell isolation kit (STEMCELL, no. 19854), according to the manufacturer’s protocol. We injected 10e4 purified B cells i.v. per mouse in Hank’s balanced salt solution 1 day prior to immunization, which resulted in an estimated engraftment of ∼500 cells per spleen given an engraftment rate of ∼5%.^110^^,^^111^^,^^112^ As recipients for VI10HL cells we used C57BL/6 mice, whereas BCR repertoire-restricted TgL mice were used as recipients for HkiL cells, to avoid anti-idiotypic rejection when analyzed more than 1 week after transfer.^58^^,^^64^^,^^71^^,^^113^

### Sample preparation and flow cytometry

We used a metal grid and a syringe plunger to mechanically disrupt spleens in order to obtain a single cell suspension of splenocytes, which we resuspended in FACS medium (RPMI [Sigma, R2405] supplemented with 5% FCS and 1% P/S). All media and buffers used for flow cytometry staining were adjusted to mouse osmolarity.^114^

Staining was performed in FACS buffer (PBS supplemented with 5% FCS) containing rat IgG (Sigma, I4131) and anti-mouse CD16/CD32 (2.4G2; BioXcell, BE0307) to block unspecific antibody binding. Additionally, we added BD Horizon brilliant stain buffer (BD Biosciences, 566385) whenever more than one brilliant violet dye was used in the staining mix. Antibodies against CD45R/B220 (RA3-6B2), Bcl-6 (K112-91), CD127 (A7R34), CD138 (281-2), CD19 (6D5), CD38 (REA616), CD4 (RM4-5), CD44 (IM7), CD45.1 (A20), CD45.2 (104), CD62L (MEL-14), CD8α (53-6.7), CXCR5 (L138D7), GL7 antigen (GL7), IgD (11-26c.2a), IgM (IL/41), Irf-4 (3E4), KLRG1 (2F1/KLRG1), and PD-1 (29F.1A12) were used for staining and purchased from BD, Miltenyi, BioLegend, and ThermoFisher Scientific (eBiosciences, Invitrogen).

MHC class II tetramers (I-A^b^) loaded with the GP_66-77_ peptide (DIYKGVYQFKSV) or the GP_415-433_ peptide (SSKAQVFEHPHIQDAASQL)^115^ were obtained from the NIH Tetramer Core Facility and were used to identify LCMV-GP- and VSV-G-specific CD4^+^ T cell responses, respectively. Tetramer staining was performed at 37°C for 1 h in FACS buffer supplemented with Dasatinib (50 nM; Sigma, CDS023389). After washing the samples with FACS buffer, we performed staining for the chemokine receptor CXCR5 for 1 h at RT. Next, samples were washed with FACS buffer followed by antibody surface staining for 30 min at 4°C. We detected LCMV-specific CD8^+^ T cell responses by using an MHC class I tetramer (H-2D^b^) loaded with the GP_33-41_ peptide (KAVYNFATC), which we obtained from the Tetramer Core Facility of the University of Lausanne. The MHC class I tetramer was combined with the antibody surface staining for 30 min at RT. Subsequent to tetramer and surface staining, cells were washed with PBS and we stained dead cells using Zombie UV Fixable Viability Kit (BioLegend, 423108) for 15 min at RT. Samples were washed with FACS buffer and fixed in 2% PFA for 10 min at RT. Samples were washed once more prior to acquisition.

We used the eBioscience Foxp3/Transcription Factor Staining Buffer Set (Invitrogen, 00-5523-00) to stain for transcription factors. After tetramer, surface, and live/dead staining we prepared the Foxp3 Fixation/Permeabilization working solution according to the manufacturer’s protocol and fixed splenocytes for 1 h at RT. After washing twice with the 1× working solution of the permeabilization buffer, we stained for transcription factors 30 min at RT. Samples were washed twice with 1× permeabilization buffer before acquisition.

Samples were acquired on a BD LSRFortessa flow cytometer (BD Biosciences), followed by analysis using FlowJo (BD Biosciences) software.

### ELISA and neutralization assay

We used an ELISA to determine serum antibody responses specific for the GP1 subunit of the LCMV WEGP or for the N119S variant (WEGP/N119S). First, we coated 96-well high-affinity binding plates with goat anti-human IgG antibody (Jackson ImmunoResearch, 109-005-098; diluted 1:500) in coating buffer (15 mM Na_2_CO_3_ and 35 mM NaHCO_3_ in ddH_2_O [pH 9.6]) at 4°C overnight. Next, the coating mix was flicked off and we added 5% milk in PBS supplemented with 0.05% Tween 20 (PBST) (Sigma, P9416) for 1 h at RT to block non-specific binding. After flicking off the blocking reagent, we added the GP1 protein or the N119S variant thereof, both coupled to a human Fc, diluted in 2.5% milk in PBST for 1 h at RT.^63^^,^^64^^,^^116^ In a separate plate we prepared 3-fold serial dilutions of serum samples or monoclonal antibodies in PBST supplemented with 2.5% milk and transferred the diluted samples into the coated and blocked ELISA plates. After 1 h of incubation at RT, plates were washed 3 times with PBST and incubated with the secondary goat anti-mouse IgG-HRP antibody (Jackson ImmunoResearch, 115-035-062; diluted 1:1,000 for use) in PBST supplemented with 2.5% milk for 1 h at RT. Plates were washed 3 times with PBST and once with PBS prior to adding the color reaction mix (0.5 mg/mL ABTS [Thermo Scientific, 34026], 28 mM citric acid, 44 mM Na_2_HPO_4_, and 0.1% H_2_O_2_ in ddH_2_O), which was incubated for 15–30 min. By adding 1% of SDS in ddH_2_O we terminated the color reaction and measured optical density (OD) at a wavelength of 405 nm on an Infinite M Plex device (TECAN).

We quantified antibody responses by either determining the serum dilution, which reached half-maxium OD (EC_50_), or we calculated the absolute antibody concentration by generating a standard curve of a monoclonal antibody and intrapolated the OD values obtained from experimental serum samples. We used naive serum to determine the background of the assay.

The KL25 antibody produced by unmutated HkiL cells binds to WEGP at high affinity (K_D_: 5 × 10e−9 M) but to WEGP/N119S at very low affinity (K_D_: <10e−5 M).^73^ Still, low-level binding to WEGP/N119S can be detected in ELISA in a concentration-dependent manner.^58^ A commonly used approach to assess affinity of a polyclonal serum antibody response to a target antigen of interest independently of specific antibody concentration consists in determining the ratio of low-affinity ligand binding to high-affinity ligand binding.^117^^,^^118^ Applying this approach to the HkiL cell antibody response to rORFV-WEGP/N119S immunization, we determined antibody titers to GP1 (high-affinity ligand) and to GP1-N119S (low-affinity ligand) as area under the curve (AUC) in separate ELISA assays. The ratio (GP1-N119S-binding AUC/GP1-binding AUC) was calculated as a surrogate of WEGP/N119S-specific serum antibody affinity, with an increase of this parameter over time indicating affinity maturation of the HkiL cell antibody response.

To determine VSV-G-specific antibody titers in serum, we performed a virus neutralization assay with VSV∗ΔG(Luc),^108^ a replication-deficient VSV pseudotyped with VSV-G and expressing the firefly luciferase as well as eGFP reporter genes. First, pre-diluted serum samples were heat-inactivated for 30 min at 56°C. Next, we performed serial dilutions of samples in 96-well U-bottom plates in MEM supplemented with 2% FCS. Samples were incubated with approximately 500 PFU of the virus for 60 min at 37°C. Next, we transferred the serum-virus mixtures into a black 96-well flat-bottom plate, added 3 × 10^4^ VeroE6 cells per well and incubated the assay for 24 h at 37°C. Next, we removed the supernatant and fixed cells with 1%–2% PFA in PBS for 10 min. The PFA was removed and PBS was added to quantify GFP-positive cells in each well using an Immunospot S6 device (C.T.L.). We determined the dilution, which resulted in 50% neutralization (NT_50_) as antibody titer.

### Immunohistochemistry

Mouse spleen tissue was fixed using the HEPES-glutamic acid buffer-mediated organic solvent protection effect (HOPE) technique (DCS Innovative, HL001R500/HL002C001), as described previously.^119^ For fluorescent staining of GL7, CD45.1, and IgD, 2 μm tissue sections were incubated with Fab fragment anti-mouse IgG (Jackson ImmunoResearch, 115-007-003) and 2.5% normal goat serum (Jackson ImmunoResearch, 005-000-121) to avoid unspecific binding. Sections were then incubated overnight with a rat anti-GL7 antibody (eBioscience, 14-5902-81), and specific binding was visualized using an appropriate species-specific Alexa Fluor 555-conjugated secondary antibody. To visualize the congenic marker CD45.1, slides were incubated in 10% mouse serum (Jackson ImmunoResearch, 015-000-120) and then stained with a mouse anti-CD45.1-FITC primary antibody (clone: A20, Invitrogen, 11-0453-85), a secondary rabbit anti-FITC antibody (Invitrogen, 71-1900) followed by incubation with a tertiary Alexa Fluor 488 donkey anti-rabbit antibody (Life Technologies, A21206). For co-staining with IgD, sections were incubated with 10% normal rat serum (Jackson ImmunoResearch, 012-000-001) to avoid unspecific bindings, followed by 1 h incubation with a rat anti-IgD Al647 conjugated (BioLegend, 405702, and Labeling kit, Life, A20186). Nuclei counterstaining was performed with DAPI (Life, D3571). Stained sections were scanned using the Panoramic 250 FLASH II (3DHISTECH) Whole Slide Scanner at a 0.221 μm/px resolution.

### Quantification and statistical analysis

For statistical testing we used GraphPad Prism software (v.10.2.3, GraphPad software). When comparing two groups, we used an unpaired two-tailed Student’s t test and for comparing two sets of two groups from one dataset we used unpaired two-tailed Student’s t tests with a Bonferroni correction. When more than two groups were compared, one-way ANOVA was performed with a Tukey’s post-test to compare all groups against each other and Šídák’s post-test to compare pre-selected groups. To analyze kinetics, we performed a two-way ANOVA or a mixed-effects analysis (when not having identical numbers of samples at each time point), both followed by Šídák’s post-test. ∗*p* values < 0.05, statistically significant; ∗∗*p* < 0.01, highly significant; and *p* ≧ 0.05, not statistically significant. Absolute numbers, when displayed on a logarithmic scale, were log-converted to obtain a near-normal distribution for statistical analysis.

## Data availability

Raw data of the experimental results reported in this study have been deposited with Zenodo and are publicly available as of the date of publication under https://doi.org/10.5281/zenodo.15596408.

## Acknowledgments

We wish to thank Gert Zimmer for providing VSV∗ΔG(Luc), Florian Kreppel for providing rAd-WEGP, Thomas Brocker for rMVA-WEGP, the NIH Tetramer Core Facility for MHC class II tetramers, the entire experimental virology group for helpful discussions, Karsten Stauffer for excellent animal handling and care, and Cynthia Saadi and Min Lu for excellent technical assistance. This work was supported by the 10.13039/501100001711Swiss National Science Foundation (no. 310030_215043 to D.D.P.) and by the 10.13039/501100007601European Union’s Horizon 2020 research and innovation program under the Marie Sklodowska-Curie grant agreement (no. 812915 to D.D.P.).

## Author contributions

A.L.K., M.M., A.-F.M., M.D., D.M., R.A., and D.D.P. designed the experiments. A.L.K., M.M., A.-F.M., M.D., and I.W. performed the experiments. A.L.K. and D.D.P. analyzed the data. A.L.K. and D.D.P. wrote the manuscript.

## Declaration of interests

D.D.P. is a founder, consultant, and shareholder of Hookipa Pharma Inc. commercializing arenavirus-based vector technology, and he, as well as D.M., are listed as inventors on corresponding patents. R.A. and M.M. have ownership interest in Prime Vector Technologies GmbH. R.A. is an inventor and patent-holder on recombinant ORFV vector (EP16741595, EP15794491). R.A. and M.M. are inventors and patent-holders on ORFV-based SARS-CoV-2 vaccines and uses thereof (EP23730776).

## Supplemental information

Document S1. Figures S1–S4

Document S2. Article plus supplemental information
