# Supplementary material for: Orf virus-based vectors induce potent germinal center B cell, Tfh cell, and CD8+ T cell responses
Source: Mol Ther. 2025 Aug 27;33(11):5383–400. doi: 10.1016/j.ymthe.2025.08.037 (PMC12628154; doi:10.1016/j.ymthe.2025.08.037)
Supplement: Document S1. Figures S1–S4 [file mmc1.pdf]

YMTHE, Volume *33*

Supplemental Information

Orf virus-based vectors induce potent germinal center B cell, Tfh cell, and $CD8^+$ T cell responses

Anna Lena Kastner, Melanie Müller, Anna-Friederike Marx, Mirela Dimitrova, Ingrid Wagner, Doron Merkler, Ralf Amann, and Daniel D. Pinschewer

Figure S1

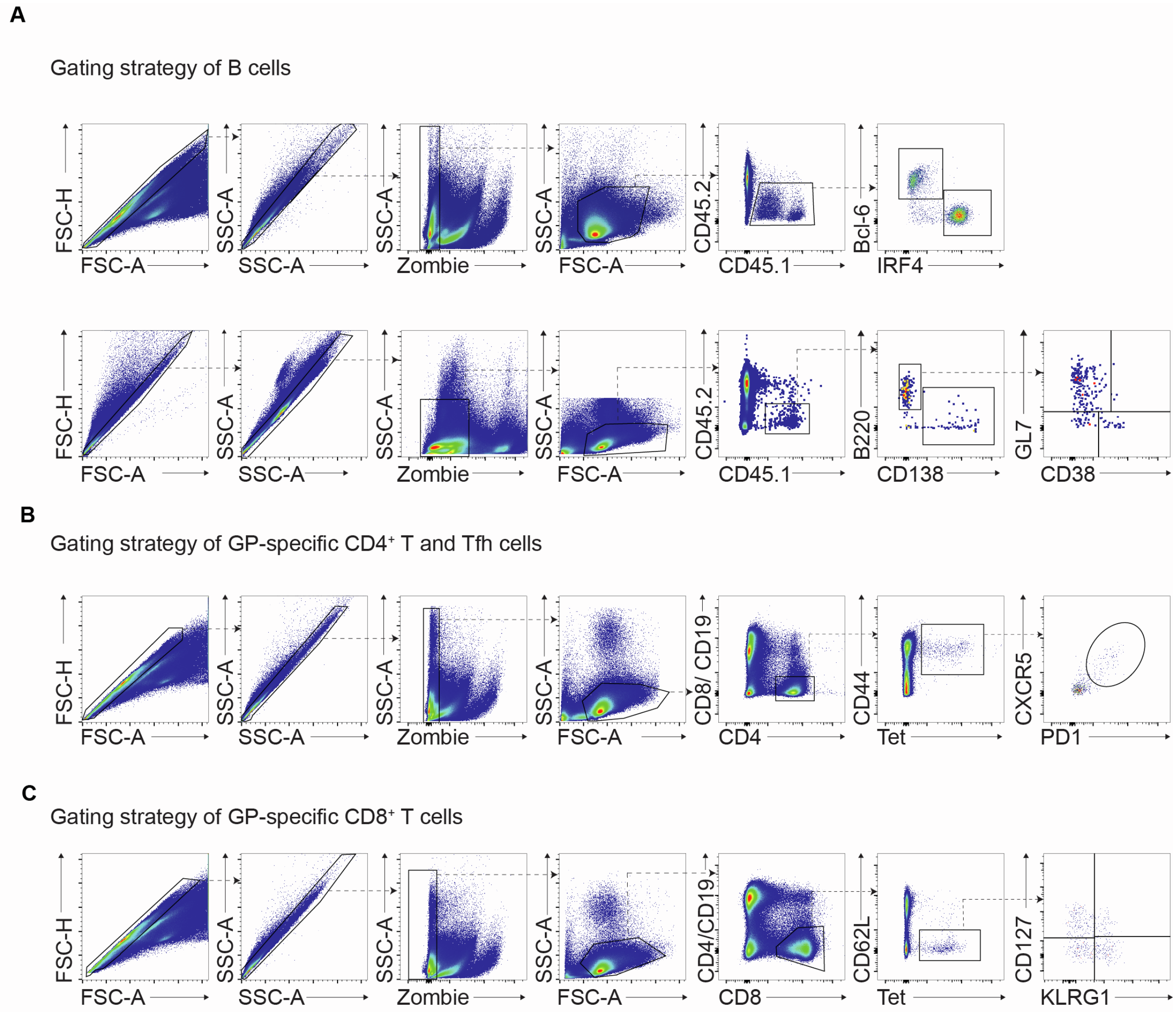

Figure S1: Flow cytometry gating strategies for the identification and characterization of CD4$^+$ and CD8$^+$ T cells and of adoptively transferred B cells.

Gating strategy for the analysis of adoptively transferred B cells (A). We used transcription factors (top row) and surface markers (bottom row) to determine the B cell phenotype. Gating strategy to determine tetramer-binding CD4$^+$ (B) and CD8$^+$ T cells (C) and their phenotype.

Figure S2

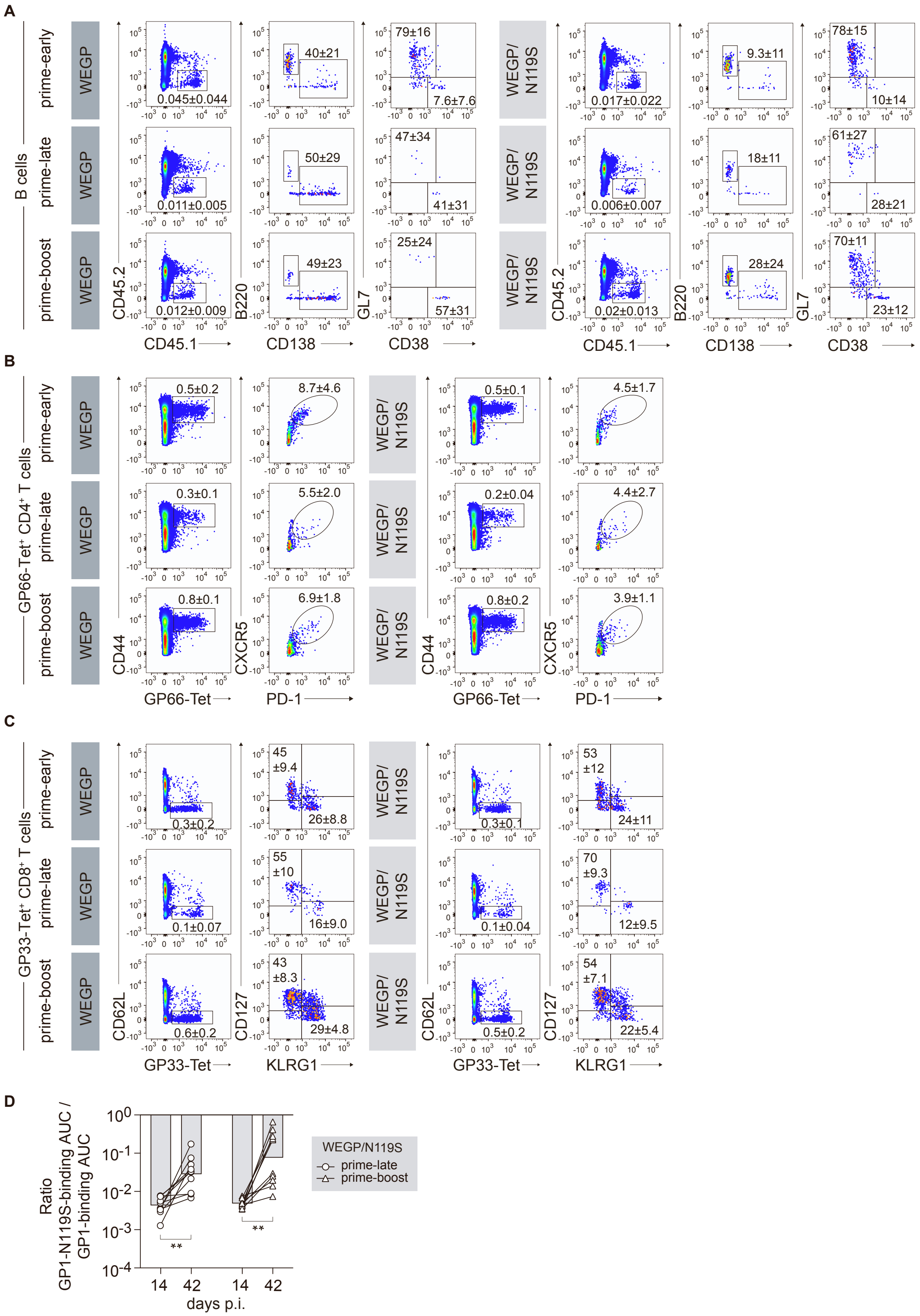
A
B cells
prime-early
prime-late
prime-boost
WEGP
WEGP/ N119S
CD45.2
CD45.1
B220
CD138
GL7
CD38
0.045±0.044
40±21
79±16
7.6±7.6
0.017±0.022
9.3±11
78±15
10±14
0.011±0.005
50±29
47±34
41±31
0.006±0.007
18±11
61±27
28±21
0.012±0.009
49±23
25±24
57±31
0.02±0.013
28±24
70±11
23±12
B
GP66-Tet+ CD4+ T cells
CD44
GP66-Tet
CXCR5
PD-1
0.5±0.2
8.7±4.6
0.5±0.1
4.5±1.7
0.3±0.1
5.5±2.0
0.2±0.04
4.4±2.7
0.8±0.1
6.9±1.8
0.8±0.2
3.9±1.1
C
GP33-Tet+ CD8+ T cells
CD62L
GP33-Tet
CD127
KLRG1
0.3±0.2
45 ±9.4
26±8.8
0.3±0.1
53 ±12
24±11
0.1±0.07
55 ±10
16±9.0
0.1±0.04
70 ±9.3
12±9.5
0.6±0.2
43 ±8.3
29±4.8
0.5±0.2
54 ±7.1
22±5.4
D
Ratio GP1-N119S-binding AUC / GP1-binding AUC
days p.i.
WEGP/N119S
prime-late
prime-boost
**
**

Figure S2: Representative FACS plots from the experiment described in Figure 2.

On d-1 we transferred HkiL B cells to six distinct groups of recipient mice of which three groups were immunized with rORFV-WEGP and three with rORFV-WEGP/N119S on d0. For each type of vaccination the first group of animals was analyzed on d14 (prime-early), whereas the second group of animals received a homologous booster immunization on d28 (prime-boost) and was analyzed on day 42 together with the third group that had only received a prime (prime-late). Representative FACS plots of HkiL B cell progeny (A; $CD45.1^{+}CD45.2^{-}$; pre-gated on live lymphocytes, see Fig. S1A) including ASCs, GC B cells and MBCs. GP66-Tet^{+} CD4 T cells (B; pre-gated on $CD4^{+}CD8^{-}CD19^{-}$ live lymphocytes, see Fig. S1B), GP66-Tet^{+} Tfh cells ($CXCR5^{+}PD1^{+}$ GP66-$Tet^{+}CD4^{+}CD8^{-}CD19^{-}$ live lymphocytes) and GP33-Tet^{+} CD8 T cells (C; left, pre-gated on $CD8^{+}CD4^{-}B220^{-}$ live lymphocytes, see Fig. S1C) as well as the $KLRG1^{+}CD127^{-}$ SLEC and $KLRG1^{-}$ $CD127^{+}$ MPEC subsets contained therein. The percentage of gated cells is indicated as mean±SD.

From the d14 and d42 sera of the rORFV-WEGP/N119S-immunized prime-late and prime-boost groups in the experiment to Fig. 2E we determined antibody titers to GP1 and to GP1-N119S, respectively, in separate ELISA assays. The ratio of GP1-N119S-binding area under the curve (AUC) to GP1-binding AUC is displayed as a surrogate of WEGP/N119S affinity, assessed independently of antibody concentration (D). Symbols represent individual mice (combined from two independent experiments) and bars indicate the group mean. Data were analysed by two-way ANOVA, followed by Šídák's post-test. *: $p<0.05$; **: $p<0.01$; ns: not statistically significant.

Figure S3

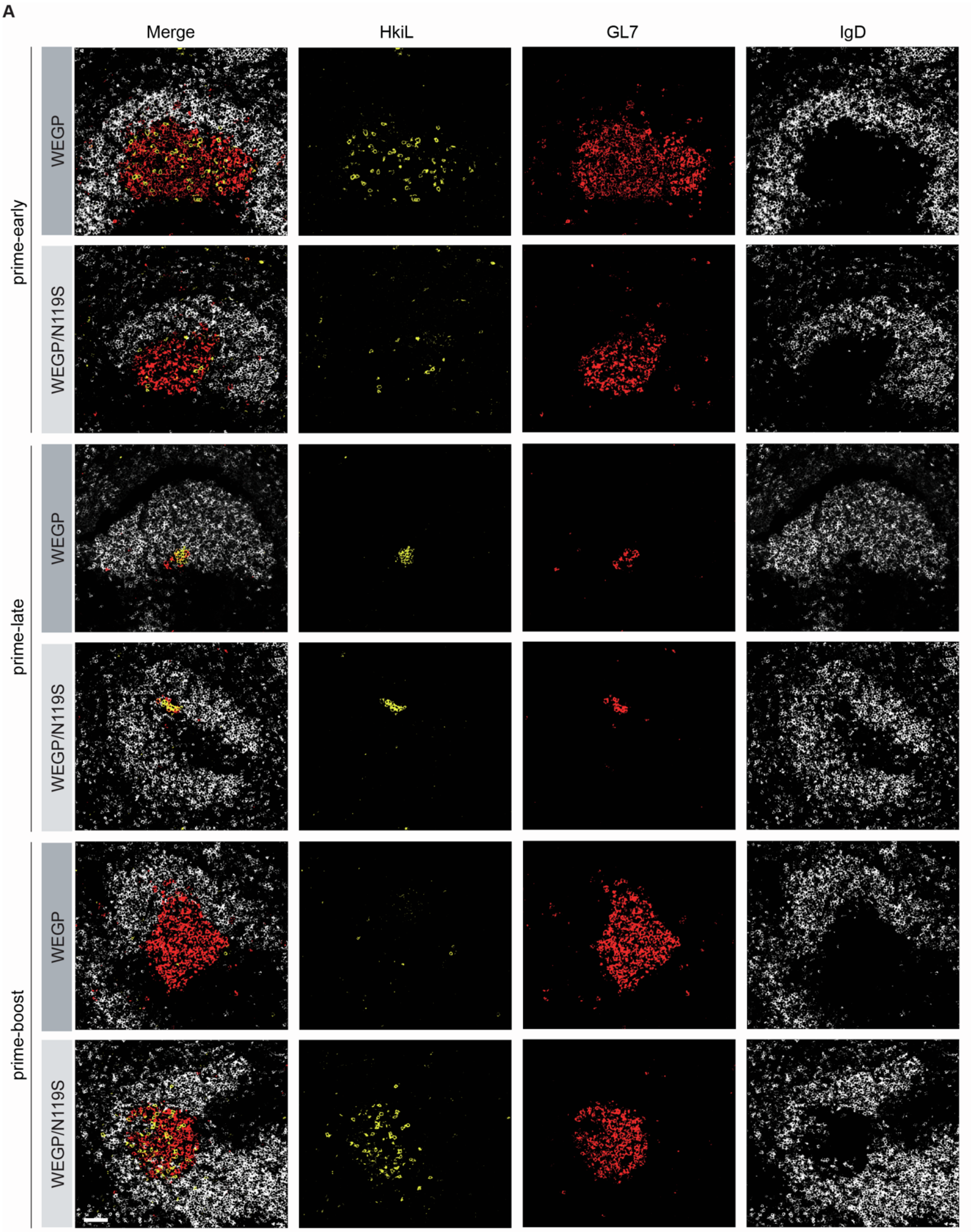
A
Merge
HkiL
GL7
IgD
prime-early
WEGP
WEGP/N119S
prime-late
WEGP
WEGP/N119S
prime-boost
WEGP
WEGP/N119S

Figure S3: Individual color layers of the immunohistochemical analysis displayed in Figure 2D.

Representative histological spleen sections of groups from Figure 2A were stained for CD45.1 (HkiL), IgD and GL7 (Magnification bar: 50 µm). The «merge» corresponds to the images shown in Fig. 2D.

Figure S4

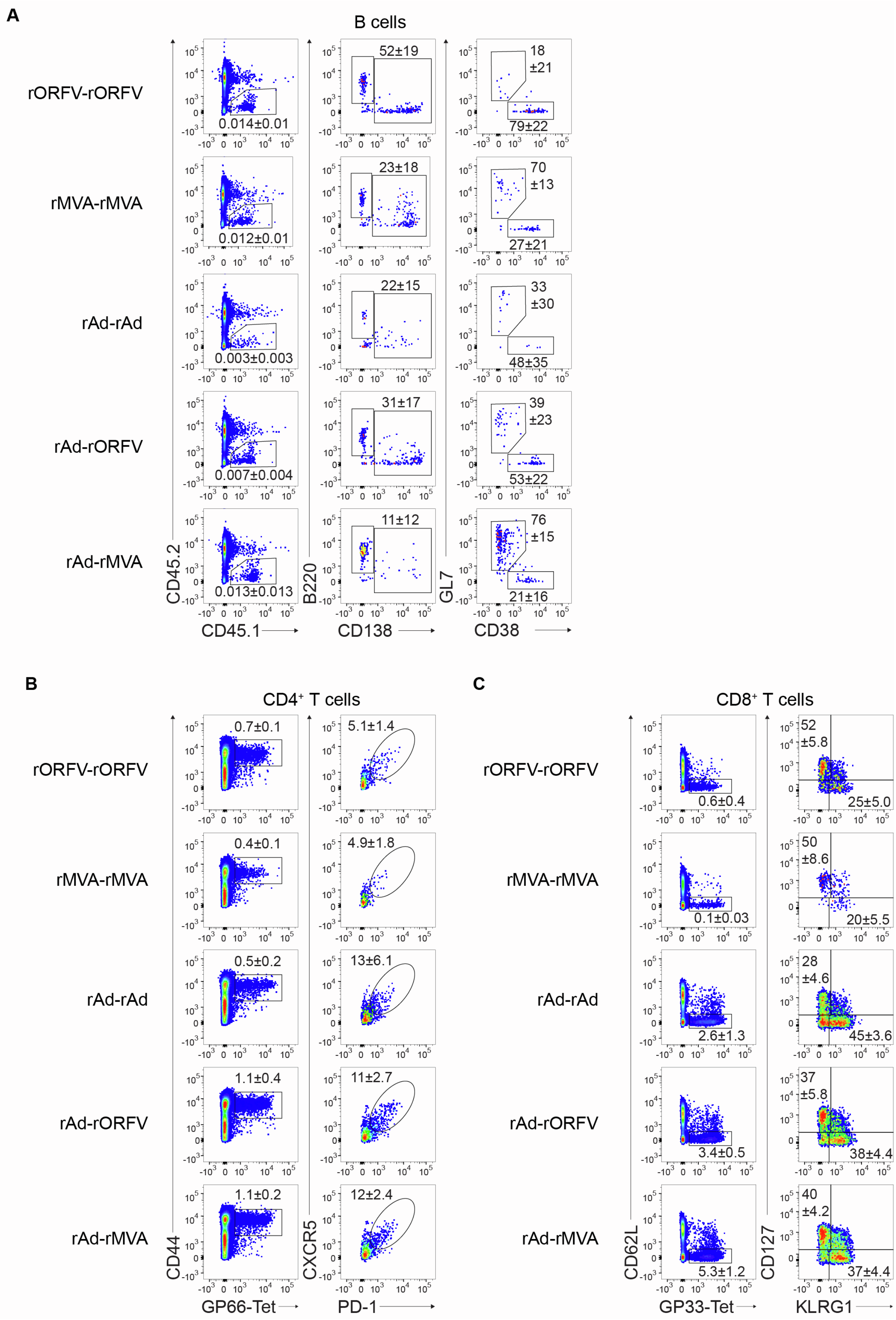
A
B cells
rORFV-rORFV
rMVA-rMVA
rAd-rAd
rAd-rORFV
rAd-rMVA
0.014±0.01
0.012±0.01
0.003±0.003
0.007±0.004
0.013±0.013
52±19
23±18
22±15
31±17
11±12
18 ±21
79±22
70 ±13
27±21
33 ±30
48±35
39 ±23
53±22
76 ±15
21±16
CD45.2
CD45.1
B220
CD138
GL7
CD38
B
CD4+ T cells
0.7±0.1
0.4±0.1
0.5±0.2
1.1±0.4
1.1±0.2
5.1±1.4
4.9±1.8
13±6.1
11±2.7
12±2.4
CD44
GP66-Tet
CXCR5
PD-1
C
CD8+ T cells
0.6±0.4
0.1±0.03
2.6±1.3
3.4±0.5
5.3±1.2
52 ±5.8
25±5.0
50 ±8.6
20±5.5
28 ±4.6
45±3.6
37 ±5.8
38±4.4
40 ±4.2
37±4.4
CD62L
GP33-Tet
CD127
KLRG1

Figure S4: Representative FACS plots from the experiment described in Figure 6.

We adoptively transferred HkiL B cells to recipient mice on d-1 and immunized them on d0 with rORFV, rMVA or rAd encoding WEGP (A). On d28 three groups of mice were boosted in a homologous manner with the respective vector. Additional two groups of rAd-WEGP-primed mice were boosted with rORFV-WEGP or rMVA-WEGP, respectively. Splenocytes were analyzed on d42 by flow cytometry. Representative FACS plots of HkiL B cell progeny (A; $CD45.1^{+}CD45.2^{-}$; pre-gated on live lymphocytes, see Fig. S1A) including ASCs, GC B cells and MBCs. GP66-Tet^{+} CD4 T cells (B; pre-gated on $CD4^{+}CD8^{-}CD19^{-}$ live lymphocytes, see Fig. S1B), GP66-Tet^{+} Tfh cells ($CXCR5^{+}PD1^{+}$ GP66-$Tet^{+}CD4^{+}CD8^{-}CD19^{-}$ live lymphocytes) and GP33-Tet^{+} CD8 T cells (C; left, pre-gated on $CD8^{+}CD4^{-}B220^{-}$ live lymphocytes, see Fig. S1C) as well as the $KLRG1^{+}CD127^{-}$ SLEC and $KLRG1^{-}CD127^{+}$ MPEC subsets contained therein. The percentage of gated cells is indicated as mean±SD.
